# Separating lymph node stations by the surgeon from the gastric cancer specimen improves the quality of nodal status evaluation

**DOI:** 10.1186/s12957-023-03146-y

**Published:** 2023-08-26

**Authors:** Aleksi Fernström, Arto Kokkola, Akseli Korpela, Pauli Puolakkainen, Johanna Louhimo

**Affiliations:** Abdominal Center, Department of Surgery, Helsinki University Hospital and University of Helsinki, Meilahti Hospital, PO Box 440, Stenbäckinkatu 9A, Helsinki, 00029 HUS Finland

**Keywords:** Gastric cancer, Nodal status, Lymph node stations, N-stage, Prognosis

## Abstract

**Background:**

In gastric cancer (GC), the pN-stage is an important prognostic factor influencing treatment. Along with the depth of invasion of the tumor, the presence of nodal metastases is one of the most important prognostic factors guiding treatment strategies in gastric cancer. Examining a small number of lymph nodes may lead to understaging of the disease; hence, it is essential for the nodal status to be precisely assessed. In this study, we explored whether dissecting lymph node stations into separate samples by the surgeon from the gastric cancer surgical specimen affects the quality of nodal status evaluation and patient outcome.

**Methods:**

The clinical data of 130 GC patients treated at the Helsinki University Hospital between 2016 and 2019 was reviewed. The performed operations included 59 total and 71 subtotal gastrectomies. The processing of the surgical specimen before the pathological examination was assessed from the operation records and pathology reports.

The association of the number of examined lymph nodes with other variables was assessed, and multivariate survival analysis was performed to explore the independent prognostic factors in disease-specific survival.

**Results:**

Dissecting lymph node stations into separate specimens before pathological evaluation yielded a significantly greater number of examined lymph nodes compared with a specimen without intervention (median 34.5 vs 21.0, *p* < 0.001). The pT-stage, the pN-stage, and the extent of lymphadenectomy were identified as independent prognostic factors, whereas dissecting the specimen’s lymph node stations did not associate with survival.

**Conclusions:**

Dissecting lymph node stations into separate specimens results in a greater number of examined lymph nodes, which has the potential to lead to a more reliable pN-stage assessment.

## Background

Gastric cancer (GC) remains the sixth most common cancer and the fourth leading cause of cancer-related deaths worldwide [1]. Its prevalence and annual incidence vary between geographic locations, with the highest age-standardized rates in East Asia, whereas in northern Europe and North America, it is relatively uncommon [1]. In 2019 in Finland, new gastric cancer cases comprised approximately 1.7% of all new cancer cases [2].

Treatment options depend mainly on the location and depth of invasion of the tumor. The only curative treatment is surgical resection. Resection strategies range from endoscopic mucosal resections to total and subtotal/distal gastrectomies with lymphadenectomy. Perioperative chemo- and chemoradiation therapies are frequently combined with surgery which has improved the survival rates of gastric cancer [3].

The most important prognostic factors of gastric cancer are the depth of invasion of the tumor and the presence of nodal metastases [4–7]. Other factors, such as the presence of distant metastasis, age, and the extent of lymphadenectomy have also been shown to affect the prognosis [4–7]. In addition, some studies have found the number of harvested lymph nodes to be an independent prognostic factor [8–10] and to affect the number of metastatic lymph nodes found [11].

Lymphadenectomy is essential in managing gastric cancer. Some studies have shown that clearing more lymph nodes may lead to more adequate staging, lower locoregional recurrence, and better survival [11–13].

Because nodal status is one of the most important factors affecting the prognosis, it is crucial for it to be adequately assessed. Examining a small number of lymph nodes may lead to understaging of the disease and therefore hinder patients from receiving appropriate care. The minimum number of excised and examined lymph nodes needed to accurately stage gastric cancer differs among guidelines, ranging from 10 to 25. Most guidelines suggest a minimum of 15 or 16 [14–26]. The AJCC TNM Staging System for gastric cancer 8th edition suggests a minimum of 16 lymph nodes, preferably over 31 [27].

Clinical practices on how the surgical specimen is processed during and after surgery vary between hospitals, and there are no international guidelines on this matter. Previously at the Helsinki University Hospital, the whole gastrectomy specimen with the perigastric tissues and lymph node stations still attached was sent en bloc for pathological evaluation. In recent years, the surgeons at the Helsinki University Hospital have developed a routine of dissecting lymph node stations into separate specimens for pathology.

The aim of this study was to determine whether dissecting lymph node stations into separate samples by the surgeon from the gastric cancer surgical specimen result in a greater number of examined lymph nodes compared with the previous en bloc approach and to explore its effect on the quality of nodal status evaluation and patient survival.

## Methods

### Data collection

In this retrospective cohort study, data from consecutive gastrectomies performed at the Helsinki University Hospital during the years 2016–2019 was obtained. A total of 130 patients were included. All patients had gastric adenocarcinoma and underwent either total or subtotal gastrectomy with curative intent and R0 resection with a formal D1 to D2 lymph node dissection. All the operations were performed by four experienced high-volume upper-gi surgeons. Patients who underwent palliative procedures, patients with tumors with histology other than adenocarcinoma and patients with distant metastasis were excluded.

The clinical data and pathologists’ reports were retrieved from the electronic patient records of our hospital. The following characteristics were recorded: sex, age at the time of procedure, the type of procedure (total or subtotal gastrectomy), the extent of lymphadenectomy, surgical technique (open or laparoscopic), the number of lymph nodes examined, number of metastatic lymph nodes found, processing of the surgical specimen by the surgeon (whether lymph node stations were dissected into separate specimens), pTNM-classification of the tumor, the location of the tumor, and possible administration of neoadjuvant therapy.

The survival data until April 2022 was obtained from the Digital and Population Data Services Agency of Finland and the cause of death data from the Statistics Finland.

### Specimen dissection and group classification

The lymphadenectomies were performed according to the Japanese gastric cancer treatment guidelines 2014 (4th edition) or 2018 (5th edition).

The preparation and processing of the surgical specimen was under the discretion of the surgeons at the time of the surgery. Due to the retrospective nature of the study, we recorded from the operation records and from the pathologist’s report the number of specimens sent to the pathologist. If five or more separate lymph node specimens were sent, we assigned the sample to the dissected specimen group, if four or less, the sample was assigned to the en bloc group.

The extent of lymphadenectomy was simplified to D1 and D2. Patients with D1, D1+, or D2-lymphadenectomies as stated by the surgeon were deemed as D1, and patients with D2 or D2+ lymphadenectomies were deemed as D2. For statistical analyses, the pT and pN classes were simplified to pT0-4 and pN0-3. Due to the small number of pT0 cases, we grouped the pT stages 0 and 1 together for the statistical analyses. The TNM classification was performed according to the 7th edition of the AJCC cancer staging manual.

### Statistical analysis

The main outcome of the study was the lymph node yield, the number of recovered lymph nodes from the surgical specimen or specimens by the pathologist. We evaluated the lymph node harvest with the actual number of lymph nodes recovered as a continuous variable, but also as a binary dichotomous variable of the recovery of 15 or less and 16 or more lymph nodes according to the AJCC recommendation. The lymph node ratio was also calculated (the number of positive nodes divided by the total number of lymph nodes).

The association of the number of harvested lymph nodes (continuous variable) with dichotomous variables was assessed with Mann–Whitney *U* test, and the association with ordinal variables were assessed with Kruskall-Wallis test. The chi-squared test (*X*^2^ test) was used to test associations between categorical variables. Logistic regression analysis was performed to compare the associations of several independent variables with the outcome of lymph node yield as a dichotomous dependent variable. Multivariate survival analysis was performed with Cox proportional hazards regression model.

A *p* value < 0.05 was considered significant in all tests. Statistical analyses were performed with the IBM SPSS statistics version 27 software.

## Results

The distribution of the demographic data of the patient cohort between the dissected specimen and en bloc groups is shown in Table 1. The only variable where a significant difference was found between the two groups was the number of examined lymph nodes.

**Table 1:** The demographic data of the patient cohort of 130 gastric cancer patients operated with curative intent and the associations of the clinical variables between the two study groups

	Dissected specimen group (*n* = 70)	En bloc group (*n* = 60)	*X*^2^ test *p* value * Mann–Whitney *U* test *p* value
Age (years)	Mean 69.9, STD 10.1	Mean 71.7, STD 10.6	*p* = 0.382*
Sex	37 males, 33 females	31 males, 29 females	*p* = 0.892
Type of gastrectomy	41 subtotal, 29 total	30 subtotal, 30 total	*p* = 0.328
Open vs laparoscopic	35 open, 35 lap	39 open, 21 lap	*p* = 0.085
Lymphadenectomy	12 D1, 58 D2	14 D1, 46 D2	*p* = 0.379
Number of examined Lnn	Median 34.5	Median 21.0	*p* < 0.001*
	IQR 25.8–48.3	IQR 15.0–31.8	
	Minimum 12	Minimum 5	
	Maximum 82	Maximum 66	
	≤ 15 Lnn 2	≤ 15 Lnn 16	*p* < 0.001
	≥ 16 Lnn 68	≥ 16 Lnn 44	
Tumor location	Proximal: 8	Proximal: 7	*p* = 0.103
	Body: 24	Body: 31	
	Distal: 35	Distal: 22	
	Several: 3	Several: 0	
Tumor size (cm)	Mean 4.3, STD 4.0	Mean 5.0, STD 4.8	*p* = 0.323*
pT	0: 4	0: 7	*p* = 0.156
	1: 19	1: 12	
	2: 10	2: 3	
	3: 25	3: 21	
	4: 12	4: 17	
pN	0: 45	0: 31	*p* = 0.160
	1: 6	1: 12	
	2: 9	2: 5	
	3: 10	3: 12	
Lymph node ratio	Mean 4.3, STD 4.0	Mean 5.0, STD 4.8	*p* = 0.190*
Neoadjuvant treatment	Yes: 35, No: 35	Yes: 21, No: 39	*p* = 0.085

The minor postoperative complications (Clavien-Dindo classification 1 and 2) could not be reliably assessed from the electronic patient records retrospectively, but complications requiring surgical, endoscopic or radiological interventions, or intensive care unit management were observed in 3 out of 26 patients with D1 (11.5%) and 22 out of 104 patients (21.2%) with D2 lymphadenectomy. The observed difference was not significant (*p* = 0.266).

The dissection of lymph node stations into separate specimens resulted in a significantly greater yield of examined lymph nodes in the whole cohort (Mann–Whitney *U* test *p* < 0.001), and moreover, in individual D1, D2, total gastrectomy, subtotal gastrectomy, laparoscopic surgery, and open surgery subgroups (Mann–Whitney *U* test *p* < 0.001).

### Factors affecting lymph node harvest

In a univariate setting, we evaluated the association of the lymph node yield between different patient groups, the number of lymph nodes as continuous variable with Mann–Whitney *U* test and as binary variable of 15 or less and 16 or more lymph nodes with *X*^2^ test.

In our cohort, the D2 lymphadenectomy did not yield a significantly greater number of examined lymph nodes compared with D1 lymphadenectomy (Mann–Whitney *U* test *p* = 0.056). However, the D2 lymphadenectomy provided significantly more specimen with 16 or more lymph nodes (*X*^2^ test *p* = 0.010). There were no significant differences observed in the number of examined lymph nodes between laparoscopic and open surgeries (Mann–Whitney *U* test *p* = 0.717, *X*^2^ test *p* = 0.523), between age groups (Kruskall-Wallis test *p* = 0.156, *X*^2^ test *p* = 0.100), sex (Mann–Whitney *U* test *p* = 0.199, *X*^2^ test *p* = 0.082), or pT-stage (Kruskall-Wallis test *p* = 0.838, *X*^2^ test *p* = 0.808).

A significant difference was observed in the number of examined lymph nodes between pN groups (Kruskall-Wallis test *p* = 0.032). A subsequent pairwise Mann–Whitney *U* test between pN groups, after the Bonferroni correction for multiple tests, revealed that a significant difference exists between pN groups 0 and 3 (*p* = 0.023). The median number of examined lymph nodes in pN group 0 was 26.0, and 39.5 in pN group 3.

Total gastrectomies yielded a significantly greater number of examined lymph nodes compared with subtotal gastrectomies (Mann–Whitney *U* test *p* = 0.007, *X*^2^ test *p* = 0.033).

We performed a multivariate Logistic Regression analysis evaluating the association of the dissection and en bloc groups on lymph node yield. The model was adjusted for the type of gastrectomy and lymphadenectomy, as well as for the surgical approach, and it revealed that dissecting the lymph node stations was the most significant independent variable predicting the recovery of at least 16 lymph nodes (Table 2).

**Table 2:** Logistic regression analysis with the outcome (dependent variable) of retrieving at least 16 lymph nodes

	*P* value	Odds ratio	95% confidence interval
En bloc vs dissected	0.001	16.28	3.29–80.45
D1 vs D2 lymphadenectomy	0.077	3.02	0.89–10.23
Subtotal vs total gastrectomy	0.062	3.47	0.94–12.79
Open vs laparoscopic	0.452	0.64	0.20–2.07

### Survival analysis

The median follow-up time was 38.5 months, with death from gastric cancer as the primary endpoint. In the survival analysis, patients alive at the end of the follow-up and deaths due to other causes were treated as censored cases (*n* = 93), one patient was lost to follow-up due to emigration. No postoperative mortality (death within 30 days from operation) was observed.

In a univariate Cox proportional hazards regression model, patient age (*p* = 0.274) or sex (*p* = 0.658), type of gastrectomy (*p* = 0.454) or lymphadenectomy (*p* = 0.492), tumor location (*p* = 0.792), or neoadjuvant treatment (*p* = 0.375) lacked association with survival in our patient material. Dissecting the lymph node stations (*p* = 0.017), surgical approach (*p* < 0.001), the recovery of at least 16 lymph nodes (*p* = 0.027), and tumor size (*p* = 0.008), as well as pT (*p* < 0.001) and pN (*p* < 0.001) stages showed significant association with survival in a univariate model. A lymph node ratio, an alternative variable to describe nodal involvement, was also significantly associated with survival (*p* < 0.001).

Table 3 shows the multivariate Cox proportional hazards regression model including the significantly associated variables. In this model, only lymph node involvement and the recovery of at least 16 lymph nodes provided independent prognostic information. It is noteworthy, that in our patient cohort, surgical approach was significantly associated with pT stage and tumor size, as well as tumor size is significantly associated with pT stage. When surgical approach and tumor size were removed from the model, pT stage emerged as a significant prognostic factor in the multivariate model (data not shown).

**Table 3:** The Cox proportional hazards multivariate regression model with the variables associated with survival with a *p* value < 0.010 in a univariate setting of Cox proportional hazards regression. Patient age or sex, type of gastrectomy or lymphadenectomy, tumor location, or neoadjuvant treatment were not associated with survival in this cohort

	Wald	*P* value	Hazard ratio	95% confidence interval
En bloc vs dissected	0.01	0.918	0.96	0.43–2.13
Recovered lymph nodes 15 or less vs 16 or more	9.87	0.002	0.18	0.06–0.53
Open vs laparoscopic	2.18	0.139	0.44	0.15–1.31
pT0-1	5.75	0.124		
pT2	0.07	0.794	1.31	0.18–9.80
pT3	1.78	0.182	3.06	0.59–15.84
pT4	3.85	0.050	5.61	1.00–31.48
pN0	15.52	0.001		
pN1	2.24	0.134	2.69	0.74–9.78
pN2	12.06	< 0.001	9.42	2.66–33.39
pN3	9.73	0.002	7.50	2.11–26.59
Tumor size ≤ 2 cm	0.53	0.913		
> 2 cm, ≤ 4 cm	0.20	0.652	0.72	0.18–2.96
> 4 cm, ≤ 6 cm	0.46	0.498	0.56	0.10–3.03
> 6 cm	0.39	0.531	0.59	0.11–3.09

## Discussion

Examining a greater number of lymph nodes improves the reliability and prognostic power of the pN-stage. This study shows that the practice of dissecting lymph node stations into separate specimens results in a significantly higher number of examined lymph nodes compared with the en bloc approach in gastric cancer surgery. This practice has already been in use in high incidence countries such as South Korea, where at first the dissection was done by surgeons, but later by a dedicated technician [28].

Several factors may affect lymph node harvest. D1 lymphadenectomy for the total gastrectomy entails 2 more lymph node stations compared with D1 for the subtotal gastrectomy and D2 lymphadenectomy entails 4 more lymph node stations for the total than for the subtotal gastrectomy [21]. Therefore, it is assumed that by definition the lymph node yield for the total gastrectomy should be greater than for the subtotal gastrectomy as is the case with D2 vs D1 lymphadenectomies as the number of removed lymph node stations is higher. Other factors such as age, tumor size, co-morbidities, and treatment in non-specialized centers have been shown to decrease lymph node harvest [12]. In addition, it has been hypothesized that the quality of the pathologic examination, the condition of the specimens, and the innate number of lymph nodes of each patient could affect lymph node harvest [13]. In our study, only the type of surgery (total vs. subtotal), and whether the lymph nodes were dissected into separate specimens were found to increase the number of examined lymph nodes.

In our cohort, a significant difference was observed in the number of examined lymph nodes between pN groups 0 and 3, and the median examined lymph nodes in pN group 3 was higher than in pN group 0. This finding suggests that examining a greater number of lymph nodes may have led to a higher pN-stage, which could indicate a possible understaging in lower pN-stages. However, it may be argued that metastatic lymph nodes are easier to discover from the tissue specimen than small normal ones. The pN-stage emerged as the strongest independent prognostic factor in disease-specific survival in our cohort.

These findings underline the importance of an adequate lymph node dissection and subsequent careful evaluation of the surgical specimen in managing gastric cancer.

Because the N-stage of the AJCC TNM classification is based solely on the number of metastatic nodes found, it is vulnerable to understaging if only a small number of lymph nodes are examined. One can also argue that the prognosis between patients with the same number of metastatic lymph nodes can differ significantly, if one of the patients had a greater number of examined lymph nodes. In recent years, other staging methods, such as the lymph node ratio-based system, have shown better prognostic power than the AJCC TNM system [29–31]. The lymph node ratio, which is the ratio of metastatic lymph nodes and the total number of examined lymph nodes, has been shown to decrease stage migration. In this system, a better prognosis is associated with lower lymph node ratios. Regardless, both the AJCC TNM classification and the lymph node ratio-based systems are arguably more reliable when the number of examined lymph nodes is high.

Dissecting lymph node stations into separate specimens showed no independent survival benefit by itself. This is acceptable because the dissection occurs after the surgical specimen is resected, thus lacking impact on the extent of the lymphadenectomy itself and its potential effects on outcome, the total number of excised lymph nodes is the same regardless of whether the surgeon does the dissection or not. Furthermore, no significant difference was found between the distribution of the number of cases in pN classes between the dissected specimen and the en bloc group, also explaining why dissecting lymph node stations into separate specimens in itself does not influence survival.

The lack of a significant difference between the number of examined lymph nodes in D2 vs. D1 lymphadenectomies in our cohort may be explained by two possible reasons. First, the proportion of D1 dissections was small in comparison with D2 dissections (26 vs. 104). Second, in the D1 group, ten of the 26 cases were extended D1 lymphadenectomies, labeled as D1+ or D1,5 or even D2− by the surgeon.

The fact that the dissection of lymph node stations into separate specimens results in such a significant increase in the number of examined lymph nodes raises the question whether the pathologists’ methods of processing surgical specimens should be revisited. The 8th edition of AJCC TNM Staging System for GC suggests a minimum of 16 lymph nodes to be excised and examined [27]. In our cohort, less than 16 lymph nodes were examined in 21 out of 130 cases; 17 out of these were cases where the specimen was sent en bloc to the pathologist. Our results imply that separating the lymph node stations of the surgical specimen by the operating surgeon may improve the lymph node yield and therefore could be adopted into routine clinical practice.

This study was a retrospective, single institution cohort study, which has its own inherent limitations. The surgical dissection and en bloc groups were not randomized although the groups were fairly similar in terms of the variables collected, as seen in Table 1. The findings of this study are based on data from a single institution, and therefore cannot be directly generalized; therefore, further studies are warranted to confirm these findings on the quality of nodal status evaluation. We propose that separating the lymph node stations would be considered in future research when studying the effect of lymphadenectomy on survival.

## Conclusions

Dissecting lymph node stations into separate specimens is an easy way to increase the number of examined lymph nodes and to improve the quality of the evaluation of lymph node status and subsequent staging of gastric cancer. This may improve the prognostic value of the pN stage, thus enhancing the quality of patient care with GC.

## Data Availability

The datasets generated and analyzed during the current study are not publicly available due to privacy, ethical or legal restrictions: GDPR restrictions of our institution by law in our country. However, they are available from the corresponding author on reasonable request.
